# Cytomegalovirus Cutaneous Infection in an Immunocompromised Patient

**DOI:** 10.7759/cureus.598

**Published:** 2016-05-03

**Authors:** Adebayo A Fasanya, Faye T Pedersen, Sulaiman Alhassan, Opoku Adjapong, Raghukumar Thirumala

**Affiliations:** 1 Pulmonary and Critical Care Medicine, Allegheny General Hospital; 2 Department of Emergency Medicine, Allegheny General Hospital; 3 Pathology, Allegheny General Hospital

**Keywords:** cmv, immunocompromised, valganciclovir, cytomegalovirus, cutaneous, skin and soft tissue infection, vesicles and bullae

## Abstract

Cytomegalovirus (CMV), a member of the Herpesviridae family, is an opportunistic infection with a typically benign course in the healthy host but has a more ominous course in the immunocompromised population. CMV infection commonly affects the visceral organs, particularly the respiratory and the gastrointestinal tract. CMV cutaneous lesions are rare and can be easily missed. We present a case of a 76-year-old woman presenting with a diffuse non-pruritic macular lesion with scattered vesicles and bullae, which was initially treated as a varicella zoster virus infection and herpes simplex viral infection, but was later found on biopsy to be due to cytomegalovirus. She has a history of Sjögren's syndrome, interstitial lung disease, and being on chronic immunosuppression therapy. This case highlights the importance of considering CMV infection in the differential diagnosis of vesicular skin lesions in immunocompromised patients.

Based on a PubMed search for “cutaneous cytomegalovirus”, “cutaneous CMV”, “cytomegalovirus skin”, and “skin CMV” in material published in the last 20 years (from 1996 to 2016) and reviewing any applicable referenced material outside of those dates, cases of cutaneous CMV are not well documented.

## Introduction

Worldwide, 70% to 90% of people are infected with cytomegalovirus (CMV) [1]. In the immunocompromised host, CMV is a common opportunistic infection that usually presents with visceral manifestations, especially in the lung, brain, eye, and gastrointestinal tract, and can lead to death [2]. Cutaneous CMV infection is relatively rare in the literature and, when reported, is often seen in patients with the human immunodeficiency virus (HIV)/acquired immunodeficiency syndrome (AIDS), or a history of solid and hematologic transplant. However, the skin may be the first site of CMV involvement [1] and can herald an ominous prognosis, so a high degree of suspicion and appropriate treatment is necessary. Cutaneous CMV has a variety of clinical presentations ranging from localized ulcers to maculopapular rashes and vesiculobullous eruptions that can mimic herpetic infections. 

## Case presentation

A 76-year-old Caucasian woman with a history of Sjögren's syndrome, interstitial lung disease with dependence on home oxygen (2 to 4 L), on chronic immunosuppression therapy (mycophenolate, prednisone, hydroxychloroquine, and rituximab), pulmonary embolus, atrial fibrillation with two prior cardioversions, and hypertension presented to the emergency department (ED) with lethargy for approximately one month and a worsening rash on her buttocks.

The rash began as two to three vesicles on her lower back approximately five months before her ED visit. Approximately one month later, the vesicles had increased in number and size. She was treated with a seven-day course of acyclovir as would be the typical treatment for a varicella zoster infection in an immunocompromised patient. She was also treated with topical steroids as well as topical nystatin when the lesions did not resolve, and no improvement was noted. In the week before her presentation in the ED, her blisters opened, and a burning pain accompanied the rash. She denied pruritus and recent fevers.

On physical exam, she had diffuse lesions with an erythematous macular base spanning over her lower back, bilateral buttocks, and the posterior part of her upper thighs with approximately 1 cm healed darkened lesions and 1 cm to 2 cm vesicles and bullae with some with minimal blood-tinged serosanguinous drainage. The lesions extended across multiple dermatomes and were non-tender to palpation. Shallow ulcers with erythema were noted on the genital labia and oral maxillary alveolar ridge. The differential diagnosis included disseminated shingles, herpes infection, fungal infection, or a drug reaction. She was placed in contact and airborne isolation due to concern for disseminated varicella zoster virus (VZV) and acyclovir was started. Informed patient consent was obtained for treatment.

**Figure 1:**
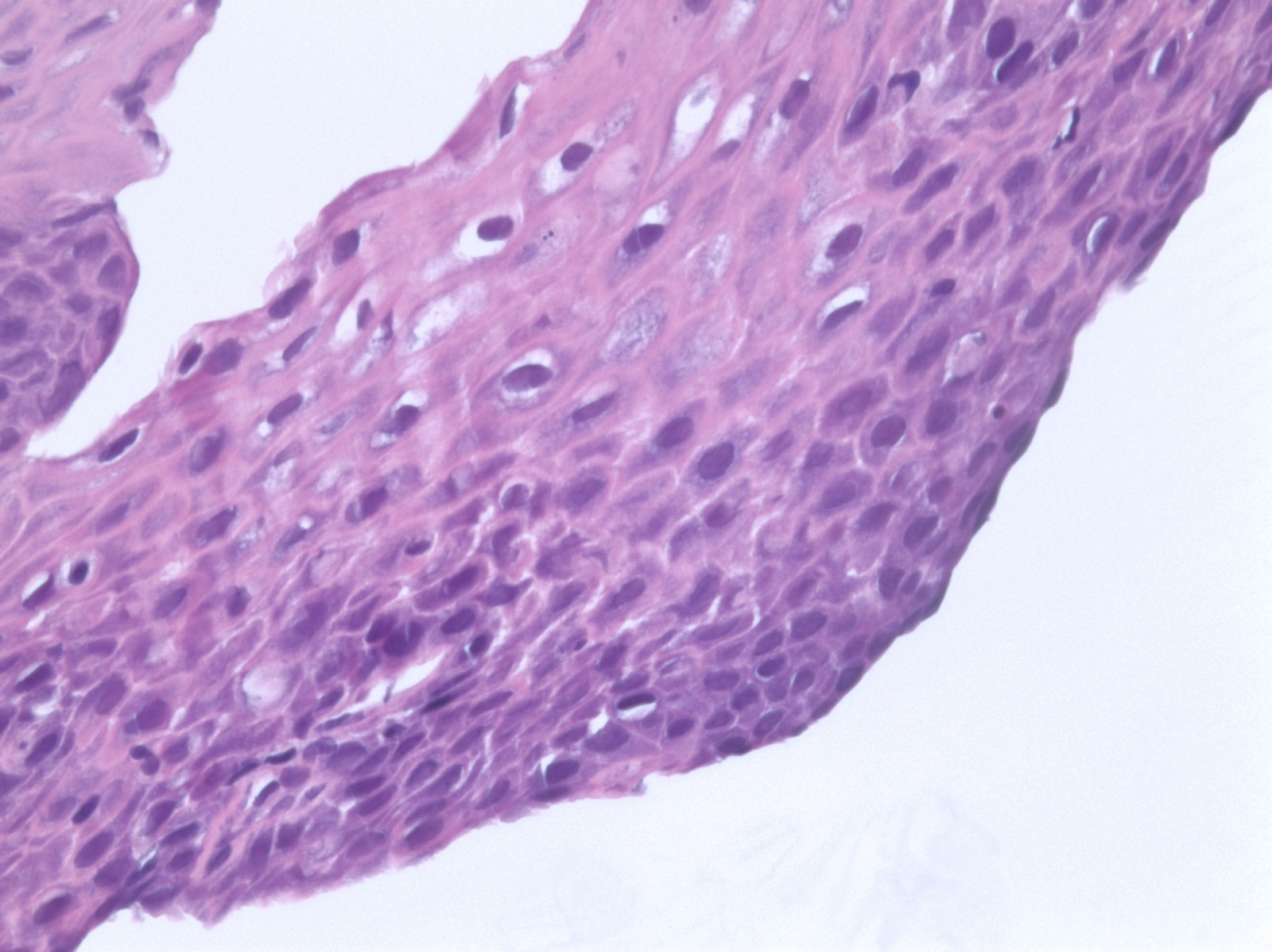
Skin punch biopsy, buttock. Cytomegalic cells with prominent intra-nuclear inclusions with surrounding halo.

A skin punch biopsy was obtained from the edge of the ulcer on her buttock. Histopathology revealed the superficial portion of the ulcer bed had the characteristic viral cytopathic changes of a CMV infection with no convincing evidence of herpes simplex virus (HSV) infection (Figure 1). 

These findings supported the diagnosis of cutaneous CMV infection. She was started on a 14-day course of valganciclovir, 450 mg twice a day, followed by daily therapy for an indefinite period due to her immunocompromised state. Her immunosuppressants were discontinued, and her prednisone dose was reduced during the 14-day initial course. At her follow-up appointment one month later, her skin lesions had resolved.

## Discussion

CMV is a member of the herpes family of DNA viruses, which includes eight separate species: HSV-1, HSV-2, VZV, Epstein-Barr virus, CMV, human herpes virus 6 (HHV-6), HHV-7, and HHV-8. A large portion of human cutaneous lesions are attributed to these Herpesviridae, and although most can be diagnosed clinically, a few will require biopsy for histologic analysis [3]. The Herpesviridae share a common architecture—a capsid with a lipid envelope enclosing a double stranded linear DNA, but the Herpesviridae rely on their host’s nucleus for DNA replication and transcription via RNA to synthesize their gene product [3]. The characteristic “owl’s eye” inclusions of CMV are found in the host’s nucleus.

Approximately 80% of adults have antibodies against CMV [4]. The acute disease of Herpesviridae is followed by an asymptomatic, quiescent state, with latency in peripheral blood leukocytes [3]. When reactivated in the skin, CMV infects the blood vessel endothelium; a biopsy can show nonspecific inflammation, possible overlying ulceration, and cytomegalic cells may contain the characteristic large, eosinophilic inclusions surrounded by a halo (the “owl’s eye” nucleus) on hematoxylin-eosin staining [3]. In the absence of these inclusions, analysis with immunohistochemical stains and molecular studies, such as *in situ* hybridization, may be used to provide a diagnosis. 

In the immunocompetent host, infection and reactivation can be followed by an asymptomatic to a mononucleosis-like course. Compromise of the host’s immune system can trigger reactivation and proliferation, and the immunosuppressed patient can present with fever, malaise, leukopenia, and the uncommon appearance of a macular rash [3].

Cutaneous CMV can have variable clinical and histological manifestations and can mimic other cutaneous infections, including VZV and HSV. Reports of cutaneous CMV are infrequent, possibly because they are self-limited [4], or due to its varied clinical presentations that are mistaken for other pathogens. The CMV lesions that affect the immunocompromised host may be specific and present as oral ulcerations, localized cutaneous lesions, crusted papules, nodules, or generalized in the form of morbilliform eruptions, verrucous lesions, perifollicular papulopustules, and urticarial and vesiculobullous eruptions [1]. Nonspecific lesions have also been noted and include maculopapular rashes, urticarial eruptions, and scarlatiniform eruptions [1].

Skin involvement with CMV can portend a disseminated infection and is associated with a mortality of 85% in six months in the immunocompromised host [5]. As such, vigilance and suspicion for CMV infection are important, especially when treatment directed against VZV and HSV does not improve the skin lesions. There are multiple regimens available for treating cutaneous CMV, including ganciclovir, foscarnet, cidofovir, and valganciclovir [4, 6]. Immunocompromised patients may require an indefinite course to minimize reactivation of the disease.

## Conclusions

Skin infection with CMV is rare with protean manifestations and can mimic herpetic infections. Vesicular skin lesions in an immunocompromised host should raise the suspicion of CMV infection. 

Given the increased risk of mortality in immunocompromised patients with CMV skin involvement, early detection with biopsy and evaluation with immunohistochemical stains and molecular studies should be considered. Further, early treatment with oral valganciclovir can successfully treat these lesions.
